# Construction Notes

**Published:** 1894-04-14

**Authors:** 


### CONSTRUCTION NOTES.

### Extension at Yeatman Hospital, Sherborne.

The committee of this hospital report that two good bedrooms have been provided by an extension of the buildings on the north side of the house on a plan submitted by Mr. Thomas Farrall. A heating apparatus has also been supplied, by which the wards, lavatories, consulting-room, matron's and nurse's sitting-rooms, bed-rooms, and passages are heated by hot-water pipes. This arrangement has worked very successfully and has added materially to the comfort of the inmates. A porcelain sink with a good supply of water has also been placed in each of the four ward lavatories.

### Falmouth Hospital.

The work of erecting the Falmouth Hospital, the generous gift of Mr. Passmore Edwards, is rapidly drawing near completion. An inspection shows that the architect, Mr. H. C. Rogers, has succeeded in designing a very compact and complete little hospital. All rooms are well arranged and within easy access. The front of the building is due south. The centre rises up handsomely to first floor and attic, and is flanked on either side and at the back on the ground floor by the main block. The main entrance is gained by means of a large hall fitted with folding doors which open into a fine corridor running from east to west of the building. The men's ward, which extends the whole length of the building, will take four beds. At the end of the corridor and adjoining the ward room there is situate the nurses' room. The part of the corridor on the left hand side of the entrance, the matron's night room, and the female ward room correspond in all respects with those just described. A room similar to the nurses' room at the east end will be used as a casual ward. The kitchen is fitted up with all possible conveniences, a scullery and larder adjoining. The water throughout the building is heated and circulated by means of a slow combustion stove. Particular attention has been paid to drainage matters.

